# Supplementary material for: Considering the Role of Behavioural Fatigue in the COVID-19 Lockdown Debates in Great Britain: A Retrospective Analysis of a National Survey Assessing the Relationships between Capability, Opportunity, Motivation, and Behaviour
Source: Behav Sci (Basel). 2024 Sep 23;14(9):852. doi: 10.3390/bs14090852 (PMC11428509; doi:10.3390/bs14090852)
Supplement: Supplementary file 1 [file behavsci-14-00852-s001.zip › behavsci-3142676-supplementary.pdf]

Supplementary Files S1.

Analysis of the dynamics of COMB components

After removing inappropriate responses, individuals who had responses to all four scales were as follows.

Day	Total answers	Complete answers	Incomplete answers
1	1652	596	1056
2	1668	601	1067
3	1619	566	1053
4	1750	591	1159
5	1671	609	1062
6	1576	568	1008
7	1665	579	1086
8	1660	604	1056
9	1687	606	1081
10	1692	574	1118
11	1768	654	1114
12	1672	614	1058
13	1677	600	1077
14	1556	610	946
15	1585	559	1026
16	1650	602	1048
17	1677	576	1101
18	1685	604	1081
19	1665	580	1085
20	1493	541	952
21	1661	576	1085
22	1633	628	1005
23	1626	589	1037
24	1656	586	1070
25	1675	662	1013
26	1464	582	882
27	1532	575	957
28	1657	652	1005
29	1678	643	1035
30	1671	635	1036

Trend modeling was carried out with seven models:

- Exponential: The reason for choosing this model is that its regression coefficient can be interpreted as an average growth rate;
- Linear: The reason for choosing this model is that its regression coefficient can be interpreted as average growth;

- Polynomials from the second to the sixth degree: the reason for choosing these models is that they are an extension of the linear model (which is a polynomial of the first degree) and are very often used for modeling in dynamics. The highest degree is sixth, as this is the maximum that can be used in MS Excel.

Different criteria are used to select the best model. The most frequently used are Adjusted R Square, AIC and BIC (Atanasov, 2018: 89-90, 177-179). The best model is that which has the largest Adjusted R Square and/or the smallest AIC and BIC.

All three criteria show that the best model for describing the dynamics of Capability is a polynomial of the third degree (See Table 1.1 in Appendix 1). All model coefficients are statistically significant (See Table 1.2 in Appendix 1). The whole period can be divided into three sub-periods:

- The first seven days, capability increases;
- From the eighth to the twenty-fourth day, capability decreases;
- From the twenty-fifth to the thirtieth day, capability increases again.

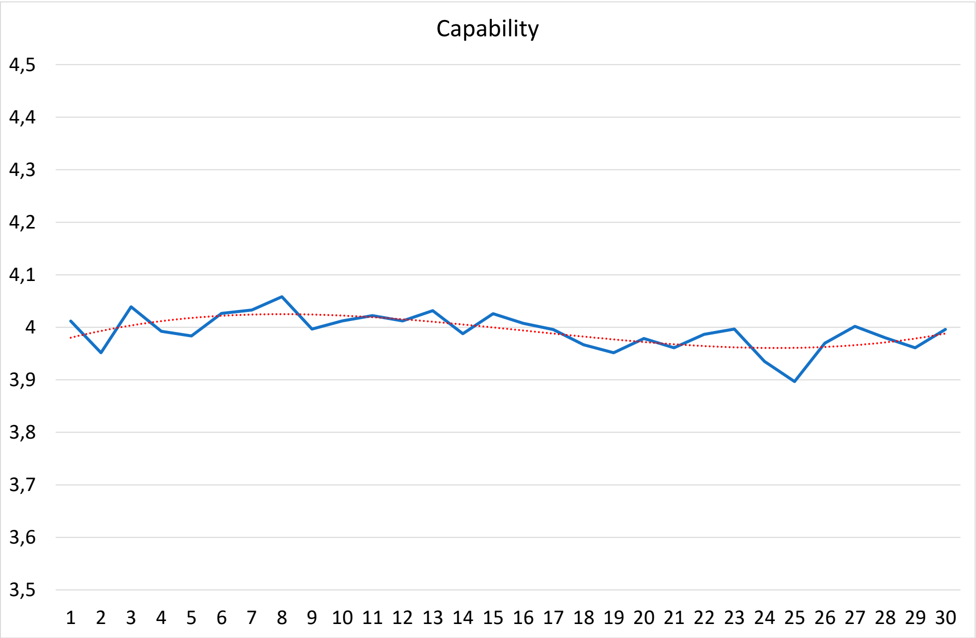

Figure S1. Dynamics of capability.

All three criteria show that the best model to describe the dynamics of Opportunity is the sixth-degree polynomial. All coefficients, except the first one, are statistically significant (see Table 1.4 in Appendix 1). The whole period can be divided into six sub-periods:

- Opportunity increases:
  - From the third to the sixth day;
  - From the twelfth to the sixteenth day;
  - On the twenty-first day;
- Opportunity decreases:

- In the first two days;
- From the seventh to the eleventh day;
- From the seventeenth to the twentieth day.

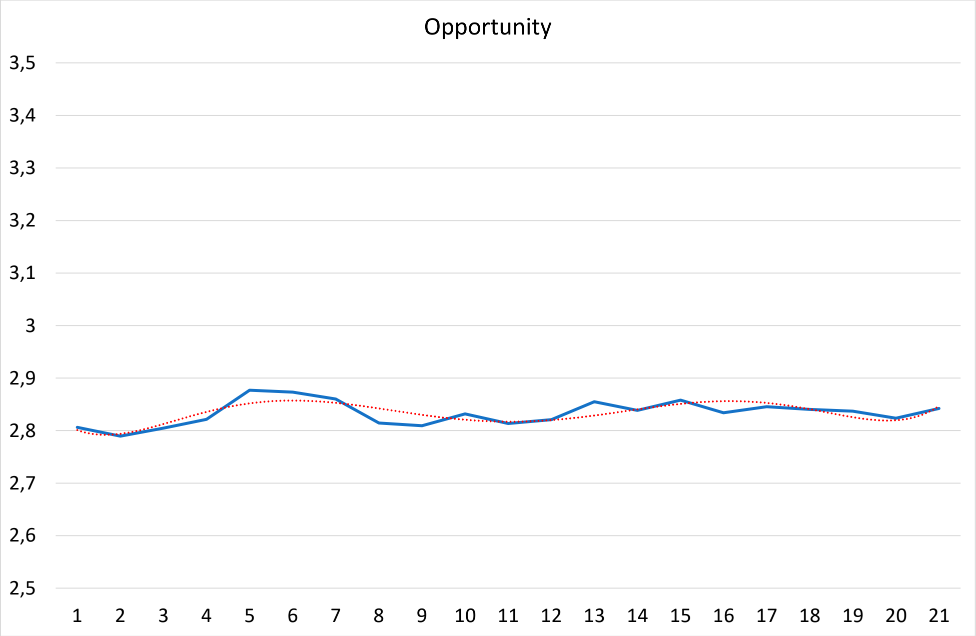

Figure S2. Dynamics of Opportunity.

According to Adjusted R Square, the best model for describing the dynamics of Motivation is the fifth-degree polynomials. AIC shows that the best model is the third-degree polynomial, and BIC shows that the best model is the linear model (see Table 1.5 in Appendix 1). When there is a discrepancy between the different criteria, it is preferable to use BIC because it is more conservative and better protects against overfitting (Atanasov, 2018: 178). Therefore, the linear model is chosen. All coefficients of this model are statistically significant (See Table 1.6 in Appendix 1). In this case, -0.0065 means that the average daily decrease is 0.0065 scores.

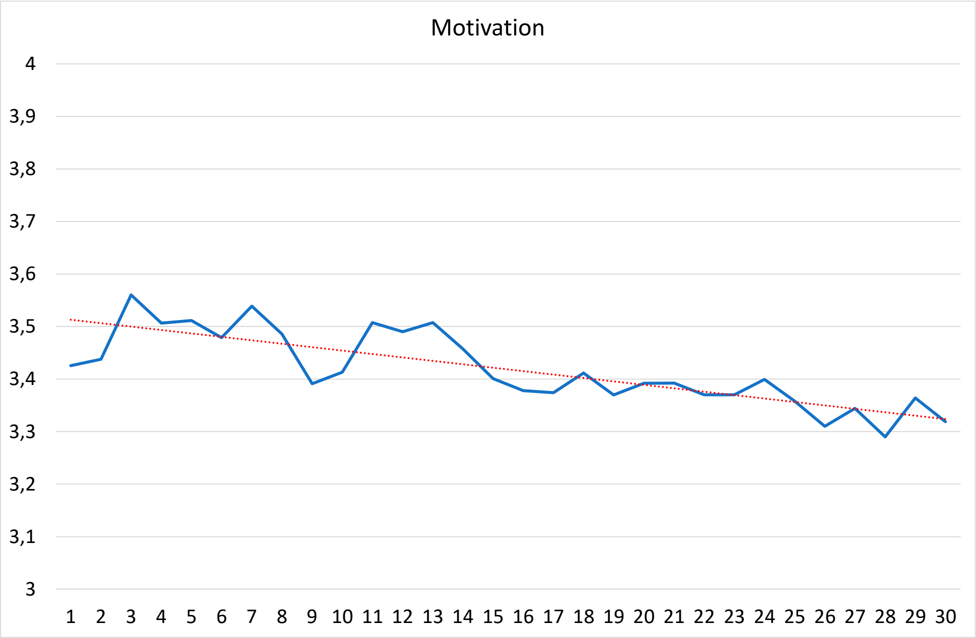

Figure S3. Dynamics of motivation.

Adjusted R Square shows that the best model to describe the dynamics of Behaviour is the fifth-degree polynomial. However, the AIC and BIC show the linear model as the best, albeit by a very small margin compared to the exponential model (see Table 1.7 in Appendix 1). Therefore, the linear model was chosen. All coefficients of this model are statistically significant (see Table 1.8 in Appendix 1). In this case, -0.0010 means that the average daily decrease is 0.0010 scores.

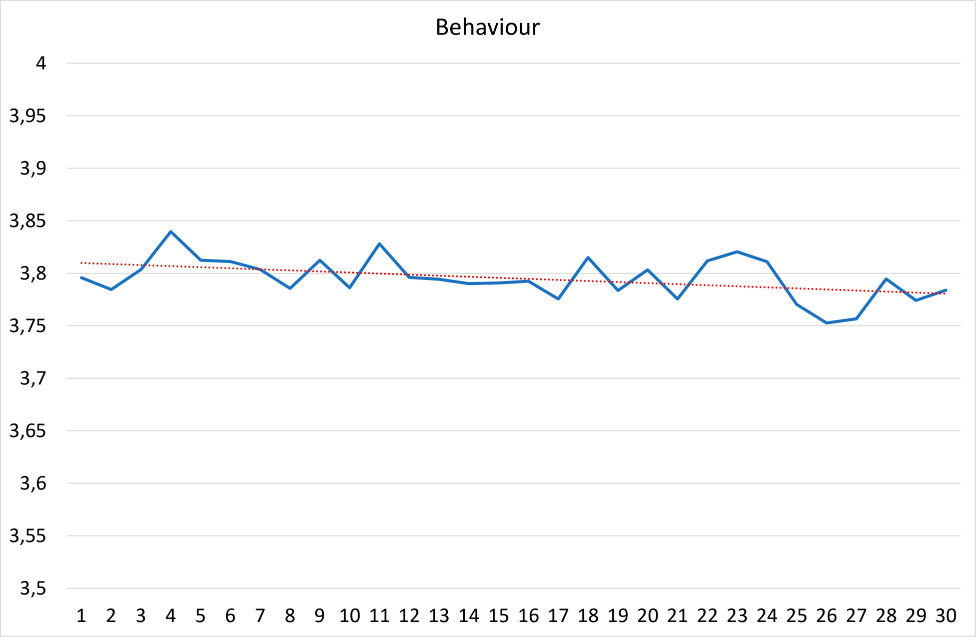

Figure S4. Dynamics of Behaviour.

Analysis of the relationships between COM-B components

Granger himself defines the simple causal two-variable model as follows (Granger, 1969: 431):

$$X_t = \sum_{j=1}^{m} a_j X_{t-j} + \sum_{j=1}^{m} b_j Y_{t-j} + \varepsilon_t$$

$$Y_t = \sum_{j=1}^{m} c_j X_{t-j} + \sum_{j=1}^{m} d_j Y_{t-j} + \eta_t$$

The definition of causality given by Grander "implies that Y_t is causing X_t provided some b_j is not zero. Similarly X_t is causing Y_t if some c_j is not zero. If both of these events occur, there is said to be a feedback relationship between X_t and Y_t." (Granger, 1969: 431)

That means that:

- If the current state of X depends on the past states of Y, but the current state of Y does not depend on the past states of X, then Y is causing X;
- If the current state of Y depends on the past states of X, but the current state of X does not depend on the past states of Y, then X is causing Y;
- If both the current state of X depends on the past state of Y, and the current state of Y depends on the past states of X, then there is mutual relationship between Y and X;
- If neither the current state of X depends on the past states of Y, nor the current state of Y depends on the past states of X, then there is no relationship between Y and X.

Granger sets the following requirements (Granger, 1969: 431):

1. X_t and Y_t must be stationary time series with zero means.
2. ε_t and η_t must be two uncorrelated white-noise series.
3. m must be finite and shorter than the given time series.

If the time series are stationary with non-zero means, then we have to include constants in the model:

$$X_t = \alpha_1 + \sum_{j=1}^{m} a_j X_{t-j} + \sum_{j=1}^{m} b_j Y_{t-j} + \varepsilon_t$$

$$Y_t = \alpha_2 + \sum_{j=1}^{m} c_j X_{t-j} + \sum_{j=1}^{m} d_j Y_{t-j} + \eta_t$$

In his Nobel lecture, Granger mentions that "standard statistical procedures […] assume data to have a property known as "stationarity." Many series in economics, particularly in finance and macroeconomics, do not have this property and can be called "integrated" or, sometimes incorrectly, "non-stationary"." (Granger, 2003: 361) "As a result, often researchers transform non-stationary time series data by first differencing to make the series stationary." (Baker and

al., 2015, 145). This means that in the case of non-stationarity, we have to use the first differences instead of the data in level form:

$$\Delta X_t = \alpha_1 + \sum_{j=1}^{m} a_j \Delta X_{t-j} + \sum_{j=1}^{m} b_j \Delta Y_{t-j} + \varepsilon_t$$

$$\Delta Y_t = \alpha_2 + \sum_{j=1}^{m} c_j \Delta X_{t-j} + \sum_{j=1}^{m} d_j \Delta Y_{t-j} + \eta_t$$

In addition, Granger continues: “It turns out that the difference between a pair of integrated series can be stationary, and this property is known as “cointegration”. [,,,] For cointegration, a pair of integrated, or smooth series, must have the property that a linear combination of them is stationary.” (Granger, 2003: 361) “Once we know that a pair of variables has the cointegration property it follows that they have a number of other interesting and useful properties. […] Further, they can be considered to be generated by what is known as the “error-correction model,” in which the change of one of the series is explained in terms of the lag of the difference between the series, possibly after scaling, and lags of the differences of each series. The other series will be represented by a similar dynamic equation. Data generated by such a model is sure to be cointegrated. The error-correction model has been particularly important in making the idea of cointegration practically useful.” (Granger, 2003: 361-362). “If a pair of series was cointegrated then at least one of them must cause the other.” (Granger, 2003: 366) That means that in the case of cointegration, we have to add error correction term (ECT) to the model:

$$\Delta X_t = \alpha_1 + \beta_1 ECT_{t-1} + \sum_{j=1}^{m} a_j \Delta X_{t-j} + \sum_{j=1}^{m} b_j \Delta Y_{t-j} + \varepsilon_t$$

$$\Delta Y_t = \alpha_2 + \beta_2 ECT_{t-1} + \sum_{j=1}^{m} c_j \Delta X_{t-j} + \sum_{j=1}^{m} d_j \Delta Y_{t-j} + \eta_t$$

These considerations lead to the following algorithm:

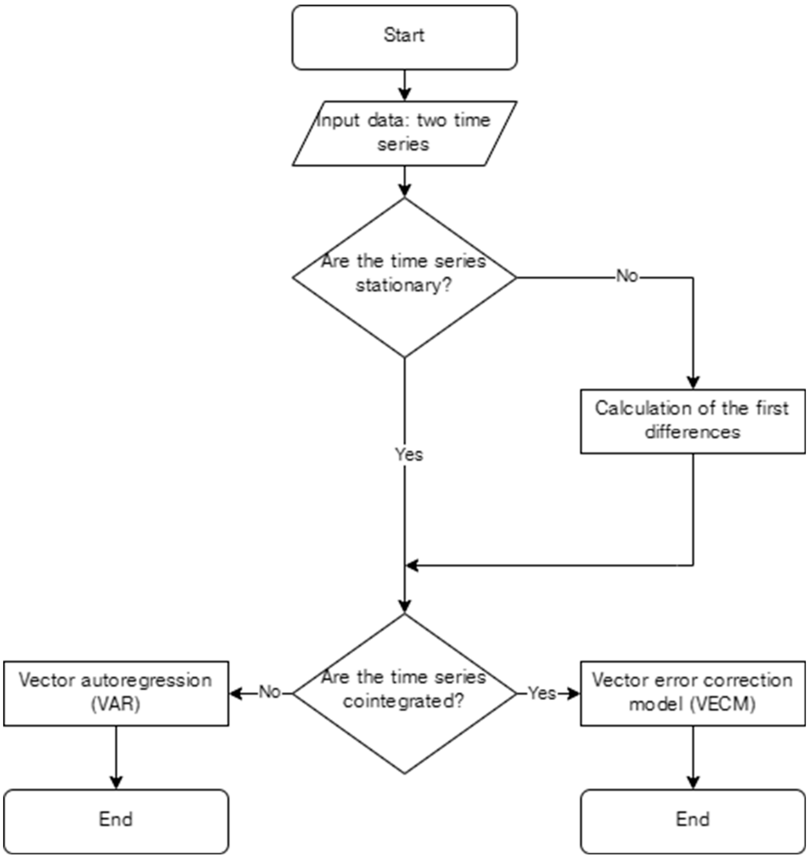

Figure S5. General algorithm of the relationships analysis.

The stationarity test was carried out with Augmented Dickey–Fuller (ADF), which is the most popular and most commonly used unit root test. With it, the null hypothesis is that the time series has a single root, i.e., the time series is non-stationary. The results show (see Table 2.1) that the time series of the data in level form of Capability ($p = 0.016$) and behavior ($p = 0.006$) do not have a unit root; i.e., they are stationary. However, the time series of Opportunity ($p = 0.116$) and motivation ($p = 0.425$) in level form have a unit root, i.e., they are non-stationary. On the other hand, time series of first differences of opportunity ($p = 0.003$) and motivation ($p = 0.000$) are stationary. Following the above algorithm, further analysis was done with time series of first differences of Opportunity and motivation. Time series of capability and behaviour will be analyzed in level form.

The cointegration test was carried out with the Johansen Cointegration Test. With it, the null hypothesis is that there is no cointegration. The results show (See Table 2.2) that in four of the pairs, there is no cointegration—capability and motivation ($p = 0.619$), Capability and Behaviour ($p = 0.758$), Opportunity and Behaviour ($p = 0.299$), and Motivation and Behaviour ($p = 0.232$)—and in the other two pairs there is cointegration—Capability and Opportunity ($p = 0.049$) and Opportunity and Motivation ($p = 0.038$). Following the above algorithm, VECM will be used for capability and Opportunity as well as for opportunity and motivation. For all other pairs, VAR will be used.

The results show that:]

- Capability affects Opportunity ($t = -3.13, p = 0.007$), but not vice versa (see Table 2.3).
- Motivation does not affect capability, nor does capability affect motivation (see Table 2.4).
- Capability affects Behaviour ($t = -2.41, p = 0.023$), but not vice versa (see Table 2.5).
- Motivation affects opportunity ($t = -3.91, p = 0.001$), but not vice versa (see Table 2.6).
- Behavior does not affect opportunity, nor does opportunity affect behaviour (see Table 2.7).
- Motivation does not affect Behaviour, nor does Behaviour affect Motivation (see Table 2.8).

The entire relationships analysis can be summarized graphically:

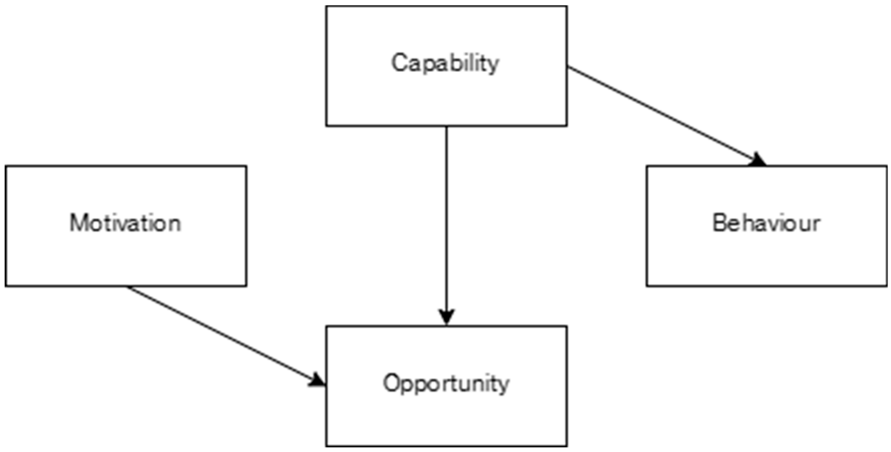

Figure S6. Statistically significant relationships.

In this diagram, the direction of the arrows indicates the directionality of the influence.

Since ECT itself is a regression model of the type $ECT_{t-1} = \gamma_0 + \gamma_1 X_{t-1} + Y_{t-1}$, it is possible to graphically represent statistically significant relationships:

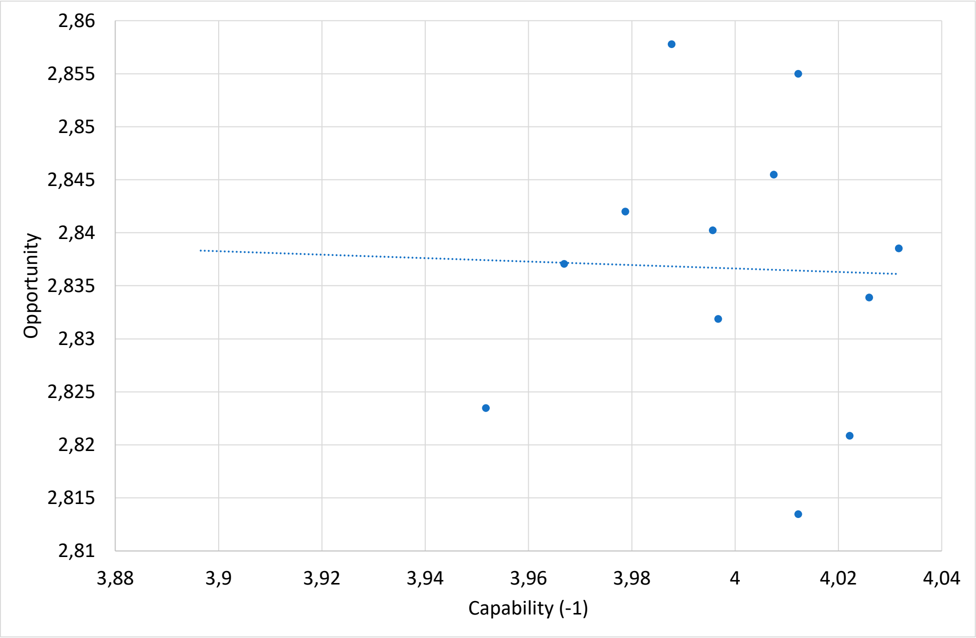

Figure S7. Statistically significant relationship between Capability and Opportunity.

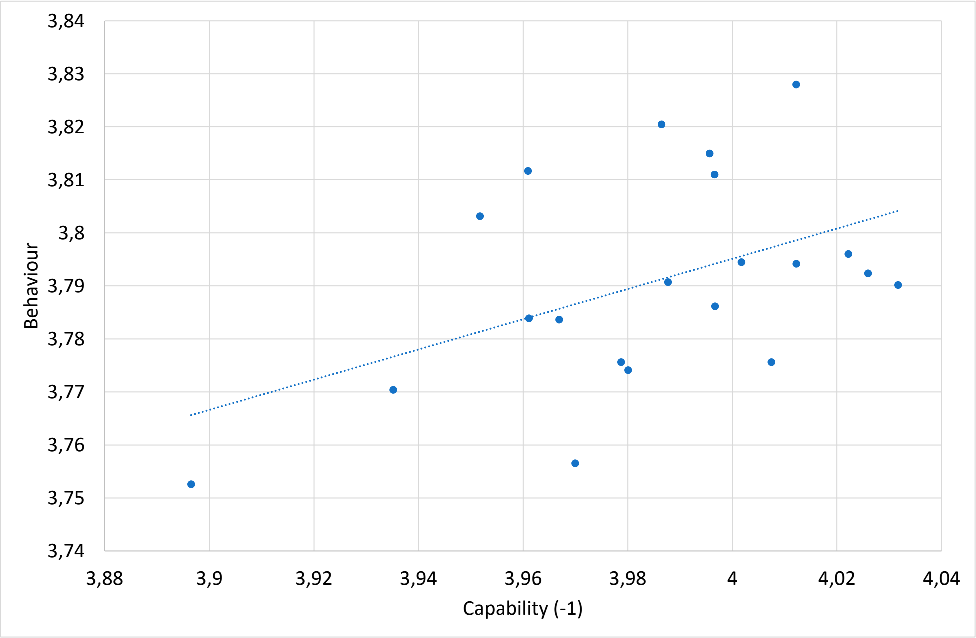

Figure S8. Statistically significant relationship between capability and behaviour.

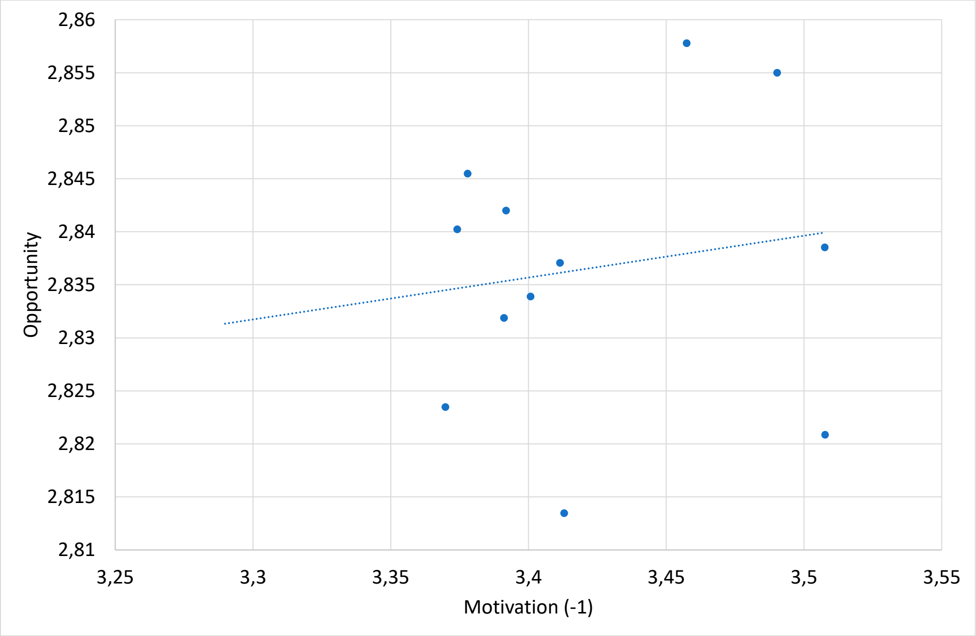

Figure S9. Statistically significant relationship between Motivation and opportunity.

Figures 7-9 allow us to determine the direction of the relationships. In the following figure, positive relationships are marked in green and negative relationships are marked in red.

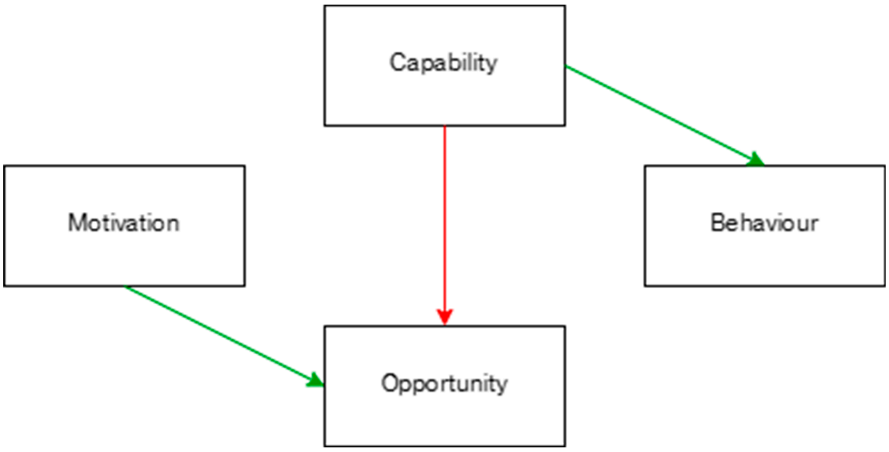

Figure S13. Statistically significant relationships.

References

Baker, D., R. Merkert, M. Kamruzzaman. 2015. Regional Aviation and Economic Growth: Cointegration and Causality Analysis in Australia. Journal of Transport Geography, No. 43, pp. 140–150

Granger, C. 2003. Time Series Analysis, Cointegration, and Applications. Nobel Lecture, December 8, pp. 360-366

Granger, C. 1969. Investigating Causal Relations by Econometric Models and Cross-spectral Methods. Econometrica, Vol. 37, No. 3, pp. 424-438

Атанасов, А. 2018. Статистически методи за анализ на динамични редове. София: Издателски комплекс – УНСС [Atanasov, A. 2018. Statisticheski metodi za analiz na dinamichni redove. Sofia: Izdatelski kompleks – UNSS]

Estimation of trends

Table 1.1. Goodness-of-fit criteria of the models (Capability).

Models	Number of independent variables	R Square	Adjusted R Square	AIC	BIC
Exponential	1	0,245	0,218	-125,04	-123,64
Linear	1	0,245	0,218	-125,04	-123,64
Polynomial	2	0,260	0,205	-123,62	-120,82
	3	0,452	**0,389**	**-130,64**	**-126,43**
	4	0,465	0,380	-129,38	-123,77
	5	0,473	0,363	-127,80	-120,80
	6	0,474	0,337	-125,88	-117,47

Table 1.2. Coefficients of the polynomial of the third degree (Capability).

Independent variables	Coefficients	t Statistics	p-value
Intercept	3,964588	178,75	**0,000**
t	0,016955	2,78	**0,010**
t^2	-0,001410	-3,11	**0,004**
t^3	0,000029	3,02	**0,006**

Table 1.3. Goodness-of-fit criteria of the models (cpportunity).

Models	Number of independent variables	R Square	Adjusted R Square	AIC	BIC
Exponential	1	0,079	0,031	-99,23	-98,18
Linear	1	0,079	0,031	-99,23	-98,19
Polynomial	2	0,152	0,057	-98,95	-96,86
	3	0,207	0,067	-98,37	-95,24
	4	0,315	0,144	-99,44	-95,26
	5	0,317	0,089	-97,50	-92,28
	6	0,626	**0,466**	**-108,17**	**-101,90**

Table 1.4. Coefficients of the polynomial of the sixth degree (Opportunity).

Independent variables	Coefficients	t Statistics	p-value
Intercept	2,86933170	55,85	**0,000**
t	-<br>0,11445969	-2,02	0,063
t^2	0,05575047	2,72	**0,017**
t^3	-<br>0,01032146	-3,09	**0,008**
t^4	0,00088656	3,28	**0,005**
t^5	-<br>0,00003567	-3,37	**0,005**

Independent variables	Coefficients	t Statistics	p-value
t^6	0,00000054	3,40	**0,004**

Table 1.5. Goodness-of-fit criteria of the models (Motivation).

Models	Number of independent variables	R Square	Adjusted R Square	AIC	BIC
Exponential	1	0,654	0,642	-104,47	-103,07
Linear	1	0,656	0,643	-104,60	**-103,20**
Polynomial	2	0,671	0,647	-104,00	-101,20
	3	0,706	0,672	**-105,32**	-101,11
	4	0,724	0,680	-105,29	-99,68
	5	0,736	**0,681**	-104,57	-97,56
	6	0,739	0,671	-102,93	-94,52

Table 1.6. Coefficients of the polynomial of the linear model (motivation).

Independent variables	Coefficients	t Statistics	p-value
Intercept	3,5196	221,80	**0,000**
t	-0,0065	-7,30	**0,000**

Table 1.7. Goodness-of-fit criteria of the models (Behaviour).

Models	Number of independent variables	R Square	Adjusted R Square	AIC	BIC
Exponential	1	0,199	0,170	-155,79	-154,39
Linear	1	0,199	0,170	**-155,79**	**-154,39**
Polynomial	2	0,220	0,162	-154,58	-151,78
	3	0,221	0,131	-152,61	-148,41
	4	0,239	0,117	-151,34	-145,73
	5	0,331	**0,191**	-153,17	-146,16
	6	0,355	0,187	-152,29	-143,88

Table 1.8. Coefficients of the linear model (Behaviour).

Independent variables	Coefficients	t Statistics	p-value
Intercept	3,8107	563,60	**0,000**
t	-0,0010	-2,64	**0,013**

Stationarity and cointegration tests, VAR and VECM

Table 2.1. Augmented Dickey–Fuller test.

Variable	Level – intercept			First differences – intercept		
	Test statistics	p-value	Lag	Test statistics	p-value	Lag
Capability	-3,48	**0,016**	0			
Opportunity	-2,57	0,116	0	-4,46	**0,003**	0
Motivation	-1,69	0,425	0	-6,49	**0,000**	0
Behaviour	-3,89	**0,006**	0			

Table 2.2. Johansen cointegration test.

Pairs of variables		Trace Statistics	p-value
Capability	Opportunity	20,35	**0,049**
Capability	Motivation	10,22	0,619
Capability	Behaviour	8,76	0,758
Opportunity	Motivation	21,09	**0,038**
Opportunity	Behaviour	13,86	0,299
Motivation	Behaviour	14,90	0,232

Table 2.3. Vector error correction model of relationship between Capability and Opportunity.

	ΔOpportunity	t Statistics	p-value	ΔCapability	t Statistics	p-value
ECT	-0,65	-3,13	**0,007**	0,003	0,27	0,788
ΔOpportunity(-1)	0,27	1,29	0,216	0,19	0,56	0,583
ΔCapability(-1)	-0,16	-1,42	0,177	-0,60	-3,13	**0,007**
Constant	0,002	0,53	0,601	-0,0001	-0,02	0,983
R-square	0,488			0,386		
Adjusted R-square	0,386			0,271		
F Statistic	4,77			3,35		
p-value	**0,016**			**0,045**		
AIC	-4,99			-4,00		
BIC	-4,79			-3,96		

Table 2.4. Vector autoregression of relationship between Capability and Motivation

	ΔMotivation	t Statistics	p-value	Capability	t Statistics	p-value
ΔMotivation(-1)	-0,23	-1,18	0,251	-0,06	-0,51	0,612

Capability(-1)	-0,17	-0,58	0,565	0,43	2,42	**0,023**
Constant	0,66	0,58	0,568	2,28	3,23	**0,004**
R-square	0,072			0,190		
Adjusted R-square	-0,002			0,125		
F Statistic	0,98			2,93		
p-value	0,391			0,072		
AIC	-3,00			-3,94		
BIC	-2,86			-3,80		

Table 2.5. Vector autoregression of relationship between Capability and behaviour

	**Behaviour**	**t Statistics**	**p-value**	**Capability**	**t Statistics**	**p-value**
Behaviour(-1)	0,17	0,97	0,343	-0,29	-0,92	0,364
Capability(-1)	0,25	2,41	**0,023**	0,43	2,35	**0,027**
Constant	2,16	3,11	**0,005**	3,39	2,74	**0,011**
R-square	0,244			0,178		
Adjusted R-square	0,186			0,115		
F Statistic	4,20			2,82		
p-value	**0,026**			0,078		
AIC	-5,08			-3,92		
BIC	-4,94			-3,78		

Table 2.6. Vector error correction model of relationship between opportunity and motivation.

	**ΔMotivation**	**t Statistics**	**p-value**	**ΔOpportunity**	**t Statistics**	**p-value**
ECT	0,02	1,18	0,256	-0,79	-3,91	**0,001**
ΔMotivation(-1)	-0,20	-0,82	0,424	-0,15	-1,89	0,078
ΔOpportunity(-1)	0,57	0,91	0,379	0,32	1,57	0,137
Constant	-0,004	-0,35	0,730	0,002	0,55	0,591
R-square	0,102			0,524		
Adjusted R-square	-0,066			0,428		
F Statistic	0,61			5,50		
p-value	0,620			**0,009**		
AIC	-2,78			-5,06		
BIC	-2,58			-4,86		

Table 2.7. Vector autoregression of relationship between Opportunity and behaviour.

	**Behaviour**	**t Statistics**	**p-value**	**ΔOpportunity**	**t Statistics**	**p-value**
Behaviour(-1)	-0,16	-0,73	0,475	0,59	1,81	0,089

ΔOpportunity(-1)	0,28	1,75	0,099	-0,13	-0,57	0,579
Constant	4,41	5,28	**0,000**	-2,23	-1,81	0,089
R-square	0,168			0,175		
Adjusted R-square	0,070			0,071		
F Statistic	1,71			1,69		
p-value	0,210			0,215		
AIC	-5,28			-4,62		
BIC	-5,13			-4,47		

Table 2.8. Vector autoregression of relationship between Motivation and Behaviour.

	**Behaviour**	**t Statistics**	**p-value**	**ΔMotivation**	**t Statistics**	**p-value**
Behaviour(-1)	0,25	1,35	0,189	0,08	0,16	0,872
ΔMotivation(-1)	0,09	1,15	0,261	-0,25	-1,27	0,215
Constant	2,83	3,96	**0,001**	-0,31	-0,17	0,870
R-square	0,122			0,061		
Adjusted R-square	0,052			-0,014		
F Statistic	1,74			0,81		
p-value	0,196			0,457		
AIC	-4,90			-2,99		
BIC	-4,75			-2,85		
